# MXene‐Induced Flexible, Water‐Retention, Semi‐Interpenetrating Network Hydrogel for Ultra‐Stable Strain Sensors with Real‐Time Gesture Recognition

**DOI:** 10.1002/advs.202303922

**Published:** 2023-09-06

**Authors:** Lianjia Zhao, Hao Xu, Lingchen Liu, Yiqiang Zheng, Wei Han, Lili Wang

**Affiliations:** ^1^ State Key Laboratory for Superlattices and Microstructures Institute of Semiconductors Chinese Academy of Sciences & Center of Materials Science and Optoelectronic Engineering University of Chinese Academy of Sciences 100083 Beijing P. R. China; ^2^ College of Physics State Key Laboratory of Inorganic Synthesis and Preparative Chemistry International Center of Future Science Jilin University 130012 Changchun P. R. China; ^3^ Qingdao Innovation and Developmemt Center of Harbin Engineering University Qingdao 266400 China

**Keywords:** machine learning, MXene hydrogel, strain sensors, ultra‐stable, water retention

## Abstract

As water‐saturated polymer networks, hydrogels are a growing family of soft materials that have recently become promising candidates for flexible electronics application. However, it remains still difficult for hydrogel‐based strain sensors to achieve the organic unity of mechanical properties, electrical conductivity, and water retention. To address this challenge, based on the template, the excellent properties of MXene nanoflakes (rich surface functional groups, high specific surface area, hydrophilicity, and conductivity) are fully utilized in this study to prepare the P(AA‐co‐AM)/MXene@PDADMAC semi‐interpenetrating network (semi‐IPN) hydrogel. The proposed hydrogel continues to exhibit excellent strain response and flexibility after 30 days of storage at room temperature, and its performance do not decrease after 1100 cycles. Considering these characteristics, a hydrogel‐based device for converting sign language into Chinese characters is successfully developed and optimized using machine learning. Therefore, this study provides novel insight and application directions for hydrogel families.

## Graphical abstract

MXene semi‐interpenetrating network hydrogels take full advantage of the characteristics of MXene nanoflakes and the interaction between components. The strain sensor based on it has high stability, high sensitivity, and fast response recovery time. And the portable device can convert sign language into Chinese characters.

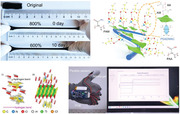

## 1 Introduction

With the rapid development of science and technology, traditional rigid electronic devices can no longer meet the developmental needs of emerging fields such as electronic skins (E‐skins),^[^ ^1^ ^]^ personalized medical monitoring,^[^ ^2^ ^]^ soft robotics,^[^ ^3^ ^]^ and human‐computer interaction.^[^ ^4^, ^5^ ^]^ Therefore, flexible electronic devices have attracted increasing attention in recent years.^[^ ^6^ ^]^ The excellent flexibility of electronic materials is key to ensuring their mechanical and electrical properties. Flexible electronic include conductive polymers (including composite conductive and intrinsically conductive polymers), carbon‐based nanomaterials (such as carbon nanotubes, graphene, and MXenes), liquid metals (such as gallium), metal nanowires (such as silver and gold nanowires), and semiconductor materials (such as silicon and germanium).^[^ ^7^ ^]^ Compared with other flexible materials, composite conductive polymers have greater potential because two parts are included: a polymer matrix and conductive fillers. Therefore, they can be considered functional materials for flexible electronic devices.

As a type of water‐saturated cross‐linked polymer network, pristine hydrogels (referring to hydrogels without conductive filler, known as P‐hydrogels) are a type of composite conductive polymer‐based material that has been widely studied in recent years. Compared to other base materials, P‐hydrogels obtain unique properties that common polymers do not possess. This is achieved by changing the types of monomer materials and polymerization conditions, including, albeit not limited to, adhesion, self‐healing, and network structure. Therefore, various P‐hydrogels can be prepared according to practical requirements. However, P‐hydrogels lack charge carriers and/or mobile charges, and their conductivity predominantly depends on water absorption, which reduces their conductivity.^[^ ^8^ ^]^ Moreover, P‐hydrogels do not generally retain water. After losing water, both conductivity and also inherent characteristics, such as flexibility are lost. This significantly limits practical applications for P‐hydrogels.^[^ ^7^ ^]^ Therefore, it is necessary to prepare composite hydrogels (C‐hydrogels) with more harmonious and uniform properties, and to improve conductivity, it is necessary to identify suitable conductive filler materials as carriers. To improve stability, the interaction between the internal structure and water molecules must be strengthened to improve water retention, and to reduce hydrogel toxicity the cross‐linking agent must be improved. Therefore, suitable conductive fillers, cross‐linkers, and water retainers are critical to the preparation of effective C‐hydrogels with excellent performance.

As emerging two‐dimensional transition metal carbons and/or nitrides,^[^ ^9^, ^10^ ^]^ MXenes have been widely used to improve hydrogel performance.^[^ ^7^, ^11^ ^]^ However, most existing studies have solely used MXenes as conductive fillers without fully exploiting their hydrophilicity, high specific surface area, multiple active sites, and abundant surface functional groups.^[^ ^11^, ^12^ ^]^ Few studies have been published using MXenes as cross‐linking agents,^[^ ^13^ ^]^ however, only single network hydrogels were constructed, resulting in a relatively single performance and low tunability compared with semi‐IPN (semi‐interpenetrating network) or IPN hydrogel.^[^ ^14^ ^]^ Therefore, constructing a semi‐IPN or IPN hydrogel that fully utilizes the characteristics of MXenes is a feasible solution for preparing C‐hydrogels with excellent performance. In this study, we used MXene@PDADMAC (where PDADMAC refers to Poly(diallyldimethylammonium chloride)) as a template, selected common AA (acrylic acid) and AM (acrylamide) as polymer monomers, and prepared a P(AA‐co‐AM)/MXene@PDADMAC semi‐IPN hydrogel (referred to hereafter as MXene‐gel). Because of the characteristics of MXene nanoflakes (hydrophilicity, abundant surface functional groups, and high specific surface area), hydrogen bonding, and electrostatic interactions between the components, hydrogels exhibit excellent mechanical properties, high conductivity, and water retention. The flexible strain sensor based on the hydrogel had a sensitivity that reached 0.98, and no obvious performance degradation was observed over the 30‐day continuous and 1100 cycle tests. Finally, in light of these advantages, a machine for converting sign language into Chinese characters was developed and optimally trained using an artificial neural network (ANN), thus demonstrating the significant potential of flexible electronic material applications in a broad range of fields.

## 2 Results and Discussion

### 2.1 Water Retention Property of MXene‐Gel

**Figure 1:**
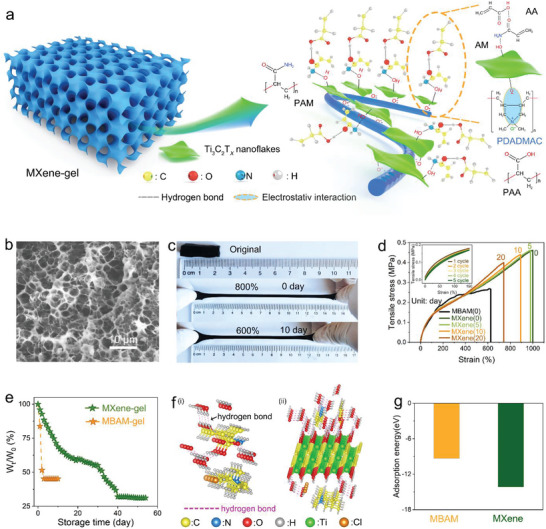
The basic properties of MXene‐gel. a) Schematic diagram of MXene‐gel composition structure. b) SEM image of lyophilized MXene‐gel. c) Optical photos of hydrogels in different stretching states. d) Mechanical properties of hydrogels at different storage days. Inset: the successive five times tensile cycle curves of MXene‐gel. e) Long‐time continuous quality monitoring curve of different hydrogels. f) Energy‐optimized geometry structure of hydrogels with water based on DFT calculation. g) The adsorption energy of different hydrogels and water by DFT calculation.

The MXene‐gel network structure is shown in **Figure** **1**a. The PAA (polyacrylic acid) and PAM (polyacrylamide) were ordered and aggregated on the MXene@PDADMAC template. For the MXene‐gel synthesis, the first is to use etchant (HCl and LiF) to etch away the Al element layer in the Ti_3_AlC_2_ MAX precursor to prepare colloidal solutions of delaminated Ti_3_C_2_T_x_ MXene nanoflakes (Figure S1a,b, Supporting Information) with abundant surface functional groups. Comparing the XRD patterns (Figure S2, Supporting Information), it can be observed that the (002) characteristic peak of Ti_3_AlC_2_ MAX shifted left from 9.4 to 6.8° after etching, and the characteristic peak of Al near 39° disappeared, indicating that Ti_3_C_2_T_x_ MXene was successfully etched.^[^ ^15^, ^16^ ^]^ Subsequently, Ti_3_C_2_T_x_ MXene and the linear cationic polymer PDADMAC^[^ ^17^ ^]^ were pre‐adsorbed in the solution through strong intermolecular electrostatic interactions, forming a MXene@PDADMAC composite linear template to ensure an orderly arrangement of Ti_3_C_2_T_x_ MXene nanoflakes. The neutral monomer AM, added later, was adsorbed on the Ti_3_C_2_T_x_ MXene nanoflakes through hydrogen bonds to achieve an orderly arrangement. The anionic monomer AA subsequently added formed a hydrogen bond with the AM monomer owing to the repulsive force between the Ti_3_C_2_T_x_ MXene nanoflakes.^[^ ^13^ ^]^ Owing to hydrogen bonding and intermolecular electrostatic interactions, before the addition of the initiator (APS), the mixed solution was finally in a stable state, in which the two monomers (AM and AA) were uniformly arranged on the MXene@PDADMAC composite linear template. Owing to the electronegativity and abundant surface functional groups of MXene, the MXene@PDADMAC composite linear template and an orderly arrangement of monomer molecules were formed, which was conducive to the formation of longer polymer chains and more stable uniform hydrogels.

Finally, after adding APS and increasing the temperature to 55 °C, the monomers were polymerized in an orderly manner. After cooling to room temperature, a flexible and stable MXene‐gel is formed. In addition to replacing the MXene colloidal solution with the traditional cross‐linking agent, MBAM powder, the preparation conditions of the control group P(AA‐co‐AM)@PDADMAC hydrogel (referred to as MBAM‐gel) were identical to those of the MXene‐gel. Comparing the SEM images of the MXene‐gel in different states, in the normal hydrated state (Figure S1c, Supporting Information), the MXene‐gel appeared in a dense state and could therefore be restored to its initial state within a suitable strain range.

The existence of Ti_3_C_2_T_x_ MXene nanoflakes can be clearly recognized (Figure S1c,d, Supporting Information), which demonstrates that Ti_3_C_2_T_x_ MXene not only acts as a cross‐linking agent but also as a conductive filler. This guarantees that the MXene‐gel has high conductivity. In the lyophilized state (Figure 1b), the hydrogel completely lost water and voids originally filled with water were completely exposed. The MXene‐gel was mainly composed of carbon (Figure S3a, Supporting Information) and oxygen (Figure S3b, Supporting Information), which were determined by the monomer molecules AA and AM. Nitrogen (Figure S3c, Supporting Information) was introduced using PAM and PDADMAC, chlorine (Figure S3e) using PDADMAC, and sulfur (Figure S3f, Supporting Information) using APS. The uniform distribution of Ti (Figure S3d, Supporting Information) confirms the uniform distribution of Ti_3_C_2_T_x_ MXene nanosheets in the hydrogel network, which is also consistent with the TEM image of the lyophilized hydrogel (Figure S1d, Supporting Information). Further observation of the lyophilized hydrogels SEM image revealed that the MXene‐gel does not produce a transparent pore structure, as reported in the literature, but a honeycomb‐like cavity structure. This proved that the AA and AM monomers were polymerized on the MXene@PDADMAC template to form the semi‐IPN hydrogel. This structure takes full advantage of the conductivity and hydrophilicity of Ti_3_C_2_T_x_ MXene: from one perspective, it assists in building the conductive network connecting the entire hydrogel, and the high conductivity of MXene fundamentally improves hydrogel conductivity; additionally, the hydrophilic nature of MXene reduces water loss, which fundamentally improves the hydrogel usefulness.

The toughness and hysteresis of the hydrogels are the physical properties that have an important impact on their performance.^[^ ^18^ ^]^ Therefore, the mechanical properties of different hydrogels were evaluated through tensile tests. First, as shown in Figure 1c, the stretching rate of the original MXene‐gel is ≈800%. After storing MXene‐gel for 10 days, the stretching rate of it still stretches up to 600%. This indicates that MXene‐gel has excellent long‐term stability, which can be further verified from mechanical property tests. As shown in Figure 1d, the tensile strength and break strain of the original MXene‐gel are 0.48 MPa and 1000% (Figure S4, Supporting Information), respectively. The mechanical properties did decay after different days of storage, but even after 20 days of storage, the tensile strength and break strain of MXene‐gel (0.4 MPa, 750%) were much higher than those of the original MBAM‐gel (0.25 MPa, 625%). These results show that compared with MBAM‐gel, MXene‐gel has better flexibility, indicating that the latter has better tensile application potential. At the same time, the fatigue resistance and hysteresis of different hydrogels were further measured by continuous loading‐unloading cycle test without rest between the two tests (Figure 1d inset; Figure S5, Supporting Information). Hysteresis refers to the misalignment degree of the loading/unloading curves between forward travel and reverse travel of the hydrogels. It becomes apparent that areas of hysteresis loops of hydrogel remained mostly constant for the following successive loading‐unloading cycles after the first cycle, indicating unchanged dissipated energies. The hysteresis error calculation equation adopted in this paper is as follows:

(1) $$\gamma_{H}=\frac{\Delta H_{max}}{Y_{FS}}\;\times 100\%$$

in which γ_*H*_ represents the hysteresis error of the hydrogels, Δ*H_max_* represents the maximum difference between the loading and unloading curves, and *Y_FS_* is tensile strengths.^[^ ^19^ ^]^ It is worth noting that the hysteresis of MXene‐gel (5.26%) is significantly smaller than that of MBAM‐gel (17.76%), indicating that it has higher stability and durability. This is very important for the application of hydrogels. The successive three times compression cycle tests (Figure S6a,b, Supporting Information) showed similar results. In addition to its tensile properties, MXene‐gel also has good shape adaptability and twistability (Figure S7, Supporting Information). At the same time, MXene‐gel has certain self‐healing properties. It can be seen from Figure S8 (Supporting Information) that after cutting the MXene‐gel, the hydrogel is self‐healing after only 12 h of reclosing the fractured surfaces together. Otherwise, no additional conditions are required. Therefore, MXene‐gel is the main research material for subsequent performance testing and characterization.

As a cross‐linked polymer network saturated with water, the properties of hydrogels are closely related to their water content.^[^ ^20^ ^]^ The excellent mechanical properties of MXene‐gel after multiple days of storage in the above tests are closely related to its water retention. Therefore, in addition to mechanical properties, the water retention of hydrogels is also a performance that must be investigated. To verify this property, we performed a series of characterizations of different hydrogels stored for different number of days. First, the specific mass loss can be seen in Figure 1e and Figure S9 (Supporting Information). The MBAM‐gel reached the maximum quality loss after 3 days, thus becoming hard and losing performance; the MXene‐gel has reached the maximum quality loss until the 40th day. This means that MXene‐gel has not only better water retention but also higher water content, which is well demonstrated by DFT theoretical simulations. As shown in Figure 1f and Figure S10 (Supporting Information), the energy‐optimized geometric structures of different hydrogels in the state of water content or not are calculated based on DFT calculation. It is obvious that no matter what kind of hydrogel, after the introduction of water molecules, one part of it has a large number of hydrogen bonds with the polymer network, and the other part still remains free. Free water will be gradually lost during the storage and use of hydrogel, thus degrading its performance. Therefore, more hydrogen bonds mean that hydrogels can produce more binding to water molecules, thus fundamentally improving the water retention of hydrogels to improve their practicability. In order to further quantify the binding of water molecules, as shown in Figure 1g, the adsorption energies between different polymer networks and water molecules are calculated. The adsorption energy of MXene‐gel (−14.16 eV) is 51.28% higher than that of MBAM‐gel (−9.36 eV), which theoretically proves that MXene‐gel has better water retention.^[^ ^21^ ^]^

### 2.2 Structural Characteristics of MXene‐Gel

**Figure 2:**
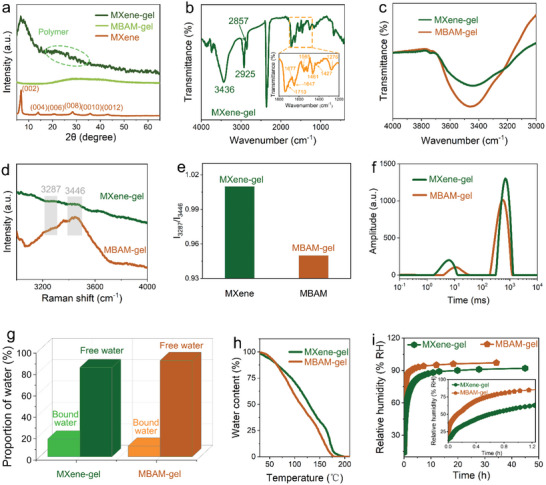
Interaction between structure and water in different hydrogels. a) XRD patterns of pristine MXene and different hydrogels. b) FTIR spectrum of lyophilized MXene‐gel. c) Comparison of FTIR spectra of different original hydrated hydrogels. d) Raman spectra and e) intensity ratio of 3287 and 3446 cm^−1^ vibration bands (*I*_3287_/*I*_3446_) of different hydrogels. f) LF‐NMR T_2_ relaxation time inversion spectra and g) proportion of different water of the prepared hydrogels. h) TGA of different hydrogels. i) Comparison of water flow stall rate of different hydrogels under sealed environment.

A series of relevant physical characterizations were performed to verify the MXene‐gel water retention. By comparing the XRD patterns of pristine Ti_3_C_2_T_x_ MXene and MXene‐gel (**Figure** **2**a), the (002) characteristic peak near 2*θ* = 6.8° of the hydrogel continues to show high intensity, and the peak (004) to (0012) could also be observed. The presence of these characteristic peaks indicates the presence of delaminated Ti_3_C_2_T_x_ MXene rather than oxides in the hydrogel, which ensures MXene‐gel conductivity.^[^ ^22^ ^]^ By comparing the XRD patterns of different hydrogels, it was identified that the broad peak at ≈2*θ* = 20° is a characteristic peak associated with polymers. Moreover, compared to the MBAM‐gel, the MXene‐gel solely consisted of additional MXene nanoflakes without other materials. However, because of the low crystallinity of the polymers, XRD cannot fully distinguish the specific polymer species contained in the hydrogel.^[^ ^23^ ^]^

To thoroughly analyze the hydrogel polymer components, the lyophilized MXene‐gel was tested using FTIR spectroscopy (Figure 2b). Sharp absorption bands at 2925 and 2857 cm^−1^ were assigned to the stretching vibrations of C─H bonds, widely present in various polymers. The bands at 1710 and 1565 cm^−1^ can be ascribed to the stretching vibrations of C═O bonds, which originate from the carboxyl (─COOH) of PAA.^[^ ^24^ ^]^ The featured band ≈1677 cm^−1^ is ascribed to the stretching vibrations of C═O bonds from PAM. The characteristic absorption band at 1647 and 1461 cm^−1^ correspond to the stretching vibrations of ─NR_4_^+^ and ─CH_3_ groups from PDADMAC, respectively.^[^ ^17^, ^25^ ^]^ Absorption bands at 1427 and 1276 cm^−1^ are ascribed to C─N stretching and ─NH_2_ rocking for primary amide (─CONH_2_), respectively, which are related to PAM.^[^ ^26^ ^]^ The FTIR spectra of the hydrogel in the original hydrated state are similar to those in the lyophilized state (Figure S11, Supporting Information), both of which indicate that the polymer in the MXene‐gel only contains PAA, PAM, and PDADMAC, which is also consistent with the experimental results. The XPS spectra (Figure S12, Supporting Information) were also all consistent with the FTIR spectra, exhibiting characteristic peaks of the corresponding chemical bonds. It is worth noting that, compared with the XPS survey spectrum of Ti_3_C_2_T_x_ MXene, there are only extremely weak characteristic peaks of titanium (456 and 563 eV) in the spectrum of MXene‐gel.^[^ ^27^ ^]^ This is because XPS mainly analyzes the surface of the material, while the Ti_3_C_2_T_x_ MXene nanoflakes are mainly coated inside the MXene‐gel,^[^ ^28^ ^]^ which is also consistent with SEM and TEM images.

In addition to identifying the kinds of polymers, FTIR spectra can also be used to analyze hydrogen bonds, which has an important contribution to the water retention of hydrogels. It is well known that for free hydroxyl groups (─OH), the sharp absorption peak is located at 3500–3700 cm^−1^. However, the formation of hydrogen bonds (O─H^…^O) increases the bond length (the bond strength decreases) of the original hydroxyl groups, which means that the characteristic peaks of the hydroxyl groups will be red‐shifted. Therefore, the stretching vibration of the hydroxyl groups at the lower wavenumber of 3100–3500 cm^−1^ means the existence of hydrogen bonding interactions.^[^ ^29^ ^]^ As shown in Figure 2b, the broad absorption band related to the stretching vibrations of the hydroxyls mainly derived from PAA, and the functional groups on the surface of MXene appears near 3436 cm^−1^, which means that the MXene‐gel contains a large number of hydrogen bonds. The hydrogen bonds of lyophilized MXene‐gel mainly come from the surface functional groups of MXene and each component, which fully enhances the mechanical properties of the MXene‐gel. As shown in Figure 2c, after MXene was selected as the cross‐linking agent, although the content of hydroxyl increased, the intensity of the broad hydroxyl vibration band became weaker. This is due to the formation of a large number of hydrogen bonds between MXene nanoflakes and polymer networks, as well as between MXene nanoflakes and water.^[^ ^30^ ^]^ This helps to fully enhance the water retention of the MXene‐gel.

In addition to FTIR spectra, Raman spectra are also used to further analyze hydrogen bonds in hydrogels. The vibration bands corresponding to hydrogen bonds are centered at 3287 and 3446 cm^−1^ (Figure 2d). These are assigned to strongly hydrogen‐bonded O─H stretching in regular tetrahedral coordination and weakly hydrogen‐bonded O─H stretching in an incomplete tetrahedral structure, respectively. The relative intensity of the two bands (*I*_3287_/*I*_3446_) can be used to estimate the ratio of strong intermolecular hydrogen bonds to weak intramolecular hydrogen bonds. As shown in Figure 2e, the I_3287_/I_3446_ ratio of MXene‐gel is higher than that of MBAM‐gel, indicating that a relatively large number of strong hydrogen bonds were formed after MXene was selected as the cross‐linking agent. This is because the surface functional groups of MXene nanoflakes form a large number of intermolecular hydrogen bonds, which also weaken the intramolecular hydrogen bonds.^[^ ^31^ ^]^ These intermolecular hydrogen bonds mean that more water molecules in the hydrogels are converted from free water to bound water, thus fundamentally improving the water retention of the hydrogel.

To measure the binding state of water in hydrogels, LF‐NMR relaxometry, a powerful tool for studying the mobility of water molecules, was used to analyze different hydrogels. LF‐NMR relaxometry can be employed to elucidate proton mobility that reflects structural heterogeneity and interactions by measuring its spin–spin relaxation time, T_2_. This important parameter can reveal the different mobility and distributions of water components in the aqueous system. Due to the chemical exchange between water protons and labile protons on the polymer and the diffusion exchange of water in different microenvironments, T_2_ of water protons in polymer solution is usually smaller than T_2_ of neat water. Therefore, a shorter T_2_ means that the water is more tightly bound to the material.^[^ ^32^ ^]^ As shown in Figure 2f, both MXene‐gel and MBAM‐gel show two proton peaks located in the 1.7–37.4 and 189–1321 ms regions, corresponding to bound water and free water, respectively. Compared with the proton peak of neat water (≈2330 ms), the T_2_ relaxation time decreases, which means that the water molecules in the hydrogels are restricted by the polymers. According to the peak area of T_2_ distribution curve, the relative ratio of bound water and free water in the hydrogels was calculated to further evaluate the binding ability between the structure of the hydrogel and water molecules.^[^ ^31^ ^]^ As shown in Figure 2g, after MXene is selected as the cross‐linking agent, the free water content decreases from 90.1% to 83.4%, while the bound water content increases from 9.9% to 16.6%. Obviously, the proportion of tightly bound water in the MXene‐gel increases significantly. Finally, the water retention of the hydrogels was verified by TGA and sealing water loss experiments. It can be seen from the TGA curves (Figure 2h) that the MBAM‐gel loses more water at the same temperature. The drum appearance of the MXene‐gel TGA curve near 150 °C is related to the presence of more bound water. This is because more energy is required for the loss of bound water. The DSC curves of MXene‐gel and MBAM‐gel (Figure S13, Supporting Information) also showed similar results. As can be seen in Figure S13 (Supporting Information), both hydrogels have two heat absorption peaks corresponding to free and bound water. Compared with MBAM‐gel, the heat absorption peaks of MXene‐gel all correspond to higher temperatures. This is a strong indication that MXene‐gel has better water retention. By placing the hydrogel in a sealed container and measuring the relative humidity of the container, it can see that the MBAM‐gel humidity rises faster and eventually gets higher (Figure 2i). This fully means that the MXene‐gel has more water retention. In conclusion, the results of FTIR, Raman, LF‐NMR, TGA and DSC can clearly show that the water interactions in MXene‐gel are stronger, so it has better water retention.

### 2.3 Strain Properties of MXene‐Gel

**Figure 3:**
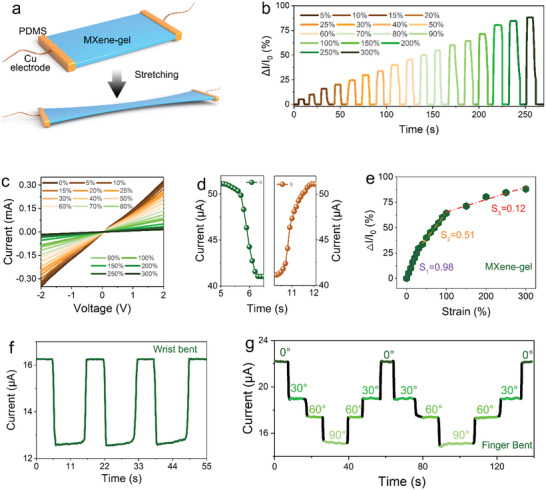
Sensing characteristics of the strain sensor. a) Diagram of the strain sensor based on MXene‐gel. b) A dynamic response curve of the strain sensor under various strain levels. c) The *I−V* curves of the strain sensor under different strain levels from 0% to 300%. d) The response and recovery times of the strain sensor under 20% strain. e) The relative current change of strain sensor under different applied strains and the slope value of current to strain. f) Detection of wrist bending. g) The dynamic response curve to the finger bending angle (0 to 90°) detected by strain sensor.

To quantify the MXene‐gel response to external strain, a strain sensor consisting of MXene‐gel as the sensing layer and Cu wires as the electrode was fabricated, as shown in **Figure** **3**a and Figure S14 (Supporting Information). Solely utilizing PDMS to encapsulate the Cu wires on both ends of the hydrogel without adding other substrate materials takes full advantage of the MXene‐gel tensile properties. As shown in Figure 3b, in the range of 0–300%, the sensor response gradually increased as the strain increased, showing the typical response of a flexible strain sensor. The step‐by‐step increase in current signal confirmed the ability of the flexible strain sensor to continuously monitor the strain over various ranges, thus ensuring accurate reliable measurement results. The I‐V test was conducted to determine the type of resistance change in the strain sensor. As shown in Figure 3c, the strain sensor follows Ohm's law and exhibits a linear relationship between voltage and current.^[^ ^33^ ^]^ Therefore, the current change in the strain sensor during subsequent tests was caused by the resistance change in the MXene‐gel. And it can be seen from Figure S15a (Supporting Information) that MXene‐gel possesses better conductivity than MBAM‐gel in the initial state.

The flexible strain sensor also exhibited fast response and recovery, ensuring timeliness of the measurement results. The measured response and recovery times were ≈49 and 66 ms, respectively (Figure 3d). At the same time, in order to express the results of the research more accurately, the strain sensitivity (S) of the sensor is defined as the slope of the current change versus strain:

(2) $$S\;=\;\delta \left(\Delta I/I_{0}\right)/\delta s$$

where Δ*I* = |*I* − *I*_0_|, *I* and *I*_0_ represent the current with and without strain respectively and *s* represents the applied strain. Based on the above standards, our sensors show different sensitivity in three different strain ranges (Figure 3e): 0.98 for 0%−30%, 0.51 for 40%−100%, and 0.12 for 100%−300%. It has been verified previously that the strain sensor based on MXene‐gel complies with Ohm's law (Figure 3c). Therefore, when the resistance of the MXene‐gel is small (that is, the stretch of the MXene‐gel is small), the resistance change of the MXene‐gel will cause a large current change, so the strain sensor has high sensitivity; on the contrary, when the resistance of the MXene‐gel is large (i.e., when the stretch of the MXene‐gel is large), the current change will decrease when the resistance changes similarly, so the device sensitivity will also decrease. Therefore, as the stretching increases, the sensitivity of our device gradually decreases. The decrease in current (increase in resistance) is attributed to changes in the conductive path in the MXene‐gel, such as the change of contact area and spacing between components.^[^ ^13^, ^34^ ^]^ When the initial stretch is small (0–30%), the MXene nanoflakes are still in close contact with each other, and the resistance change of semi‐IPN hydrogel is mainly caused by the reduction of the contact area between the polymer and the MXene nanoflakes caused by the stretching of the polymer network. As the stretching increases (40−100%), the MXene nanoflakes also begin to separate slowly. And, the sensitivity of the strain sensor also changes due to the change of the conductive path. Finally, when the MXene nanoflakes are almost completely separated from each other, the conductive path also becomes the final form, so the sensitivity gradually approaches zero. And the work still has some advantages over the hydrogel sensors that have been reported, especially in terms of water retention, response time, and recovery time (Table S1, Supporting Information). Furthermore, through the addition of flexible strain sensors, real‐time, physical signal monitoring of wrist and finger is successfully achieved, as illustrated in Figure 3f,g, which can clearly identify the movement by comparing the intensity and shape of the curves. Especially for finger bending, due to the adhesiveness of the MXene‐gel itself, continuous and accurate detection of the finger bending angle can be achieved.

### 2.4 Stability of MXene‐Gel

**Figure 4:**
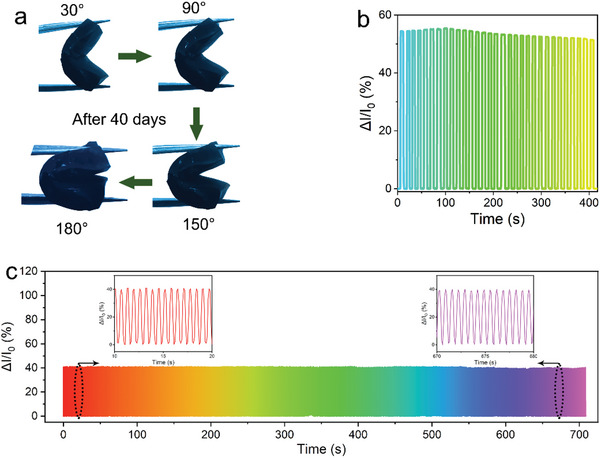
Stability characterizations of MXene‐gel. a) Optical photos of MXene‐gel bent at different angles after 40 days of storage. b) Strain dynamic response curve of MXene‐gel during a one‐month storage period. c) Working stability tested over 1100 cycles at a strain of 50%. Inset: the detailed durability performance under a strain of 50%.

Owing to its excellent water retention and strain properties, the MXene‐gel exhibits remarkable stability. The most significant challenges encountered by ordinary hydrogels are the loss of flexibility, electrical conductivity, and other properties that accompany water loss. The proposed MXene‐gel demonstrates excellent water retention and good flexibility after water loss, which extends its service life. As shown in **Figure** **4**a, the MXene‐gel remains pliable as it could be easily bent to 180° after 40 d storage. Simultaneously, it can be observed from the dynamic response test conducted over 30 consecutive days (Figure 4b) that as the weight decreased, minimal change was observed in the sensor performance. Also as can be seen in Figure S15b (Supporting Information), its current is only weakly attenuated. This is because the MXene nanoflakes enhanced the MXene‐gel conductivity and mechanical properties. Therefore, despite the loss of a certain amount of water, the flexible strain sensor based on MXene‐gel continued to exhibit excellent flexibility and strain response. These flexible strain sensors also showed good durability during cyclic testing (1100 cycles at a strain of 50%, Figure 4c); a comparison of the previous and following cycles (inset of Figure 4c) showed that the value and shape of the peaks for the electrical signals were almost unchanged, indicating that the sensor had excellent cyclic stability and a long working life. This stable sensing capability satisfies the requirements for effective long‐term detection in practical applications.

### 2.5 Application of MXene‐Gel

**Figure 5:**
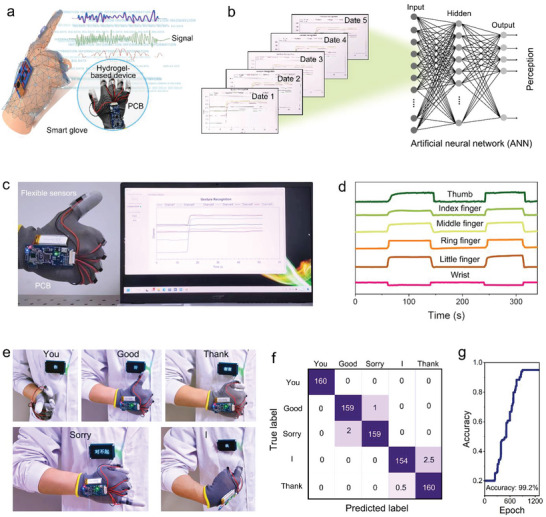
Wearable sign language‐Chinese character conversion machine based on MXene‐gel. a) Schematic illustration of the conversion system. b) Artificial neural network (ANN) general architecture with input neurons, hidden neurons, and output neurons. c) Photograph of the wearable conversion system translating sign language to Chinese characters “good” and displaying in real‐time on a commercial computer application interface. d) Voltage changes before and after bending at different parts. e) Different sign languages are converted into corresponding Chinese characters. f) The confusion matrix showing the classification of characters recognition. g) The relationship between accuracy and epoch.

Sign language uses gestures to measure actions, and simulates images or syllables based on changes in gestures, forming certain meaning or words.^[^ ^35^, ^36^, ^37^ ^]^ It is a hand‐based language that allows those with hearing impairments or an inability to speak to communicate and exchange ideas and, is therefore, an important tool for audio language.^[^ ^38^ ^]^ While this is a primary communication tool for those with hearing impairments, sign language is incomprehensible to people who do not fall into that category. Therefore, to facilitate the daily lives of hearing‐impaired people and meet their communication needs with the non‐hearing impaired population, a mobile sign‐language device would be considerably beneficial for inclusion and improved understanding. However, devices capable of this functionality are nascent in research and development in this field. From this perspective, considering the excellent sensing performance and stability of the prepared MXene‐gel, a wearable sign Language‐Chinese character conversion device was designed to address this challenge. The proposed wearable conversion system (**Figure** **5**a; Figures S16 and S17, Supporting Information) includes a sign language conversion and Chinese character display system. The former consists of a sign language acquisition and a Bluetooth transmission system. The primary component of the sign language acquisition system is a strain sensor based on MXene‐gel, which fits the epidermis of the finger in both stretching and releasing states. Hand movement are converted by sensor arrays into electrical signals, which are subsequently transmitted to the Chinese character display system using the Bluetooth transmission circuit.

In this device, the MXene‐gel behaves as a sensing system (Figure S18, Supporting Information), enabling omnidirectional monitoring of fingers and wrist bending activity. After the user forms a sign language‐based gesture, the sensing system stretches as the fingers stretch. Simultaneously, the data acquisition system collects the real‐time voltage change in the sensing system and transmits the signal to the Chinese character display system using the Bluetooth module. The Chinese character display system presents Chinese characters corresponding to gestures according to a preset voltage threshold. To improve the wearable conversion device recognition accuracy, it was trained using machine learning. As shown in Figure 5b, an ANN was constructed containing three neuronal layers (input, hidden, and output).^[^ ^39^ ^]^ As a parallel‐distributed system, it uses an entirely different mechanism than conventional information processing techniques. This overcomes the shortcomings of traditional logical artificial intelligence in processing intuitive and unstructured information with adaptive, self‐organizing, and real‐time learning properties. Different gestures are imported into the ANN, recognized, converted, and output to the display screen. When the network output does not match the desired output, the error is calculated and propagated back to the network to update the entire system.^[^ ^40^ ^]^ After several training sessions and updates, the wearable conversion system successfully recognizes various sign languages. As shown in Figure 5c, signal changes in each channel are clearly observed when the user completes a gesture “good”.

The stable operation of the entire wearable conversion system stems from the stable performance of the MXene‐gel‐based flexible strain sensors (Figure 5d). The MXene‐gel is located on the outer side of the finger. As the finger bends, the MXene‐gel stretches; therefore, the resistance increases, which increases the voltage division. The MXene‐gel is also located on the inner side of the wrist. When the wrist bands, the MXene‐gel is squeezed; therefore, resistance is reduced, which causes the voltage division to decrease. Figure 5e shows the sign language conversion results, and achieves the sign language‐to‐Chinese character conversion display of specific words such as “you,” “good,” “I,” “thank you,” and “sorry”, respectively. Accurate conversions can be achieved for these sign languages. Finally, the proposed system's classification recognition rate is tested. Figure 5f shows the classification accuracy and confusion matrix for each Chinese character. Each row in the matrix represents a true character while each column represents a predicted character. One of the five characters (You) achieved 100% classification accuracy, with an overall classification accuracy of 99.24%. The accuracy of the whole system can be further seen in Figure 5g. When the number of training sessions was low (≤600), the accuracy was low, only ≈59%; as the number of training sessions increased, the accuracy gradually increased until the accuracy was maintained at ≈99.2% after ≈1200 training sessions without further change. In conclusion, the wearable conversion machine can realize the conversion of sign language to Chinese characters, which brings great convenience to the life of the hearing‐impaired.

## 3 Conclusion

In this study, a P(AA‐co‐AM)/MXene@PDADMAC semi‐IPN hydrogel was synthesized using the template copolymerization of AA and AM in the presence of MXene@PDADMAC. In MXene‐gels, hydrogen bonds and electrostatic interactions between the components produce excellent mechanical properties, high conductivity, and water retention in hydrogels. As a cross‐linking agent, MXene nanoflakes not only act as conductive fillers to increase hydrogel conductivity but also increase hydrogel moisture retention owing to their hydrophilicity and functional groups. A series of physical characterizations and complementary experiments proved that the P(AA‐co‐AM)/MXene@PDADMAC semi‐IPN hydrogel has a unique structure (honeycomb‐like chamber structure), high strain performance (high sensitivity and stability), and excellent moisture retention (not dried for 40 d). In addition, a machine‐learning‐based hydrogel device was developed for converting sign language into Chinese characters, facilitating daily life communication between the hearing‐impaired and general population.

## 4 Experimental Section

### Synthesis of Colloidal Solutions of Delaminated Ti_3_C_2_T_x_ MXene Flakes

The Ti_3_C_2_T_x_ MXene colloidal solutions could be obtained in two steps, including the preparation of the multilayer Ti_3_C_2_T_x_ powder and the centrifugation of the aqueous dispersion of the powder. Wet etching was used to remove the Al‐element layers in the middle of Ti_3_AlC_2_ MAX to prepare multilayer Ti_3_C_2_T_x_ MXene powder. Specifically, 3 g Ti_3_AlC_2_ MAX powder was first added to a pre‐mixed etchant solution (4.8 g LiF and 60 ml of 9 m HCl) at room temperature. Then the above solution was stirred for 48 h under the condition of 35 °C oil bath. After the etching, the above solution was centrifuged at 3500 rpm for 5 min, and the obtained precipitate was stirred in 60 mL of 2 m H_2_SO_4_ for 30 min to remove the remaining etchant. Afterward, the precipitate was continuously washed with deionized (DI) water and centrifuged until the supernatant is close to neutral. At this time, the precipitate was the required multilayer Ti_3_C_2_T_x_ MXene powder. The Ti_3_C_2_T_x_ MXene powder was dissolved in 75 ml of DI water, centrifuged at 3500 rpm for 1 h, and the supernatant was the colloidal solution of delaminated Ti_3_C_2_T_x_ MXene flakes, which was referred to as the Ti_3_C_2_T_x_ MXene colloidal solution.

### Synthesis of Semi‐IPN Network Hydrogel and Flexible Strain Sensor Based on Cross‐Linked poly(AA‐co‐AM)/MXene@PDADMAC

The MXene‐gel was produced by heating and copolymerization. Add 5 mL of Ti_3_C_2_T_x_ MXene colloidal solution (≈8.5 mg·mL^−1^), 1 g of AM powder, 4 mL of AA solution, 0.1 g of APS powder, and 10 mL of DI water to the 2 mL of PDADMAC solution in sequence. Then, the uniformly mixed solution was transferred to the mold, wrapped in tin foil, and then placed in an oven at 55 °C for heating for 12 h. After naturally cooling to room temperature, the hydrogel was peeled from the mold for subsequent experiments. Other materials and conditions remained unchanged, 5 mg of traditional cross‐linking agent MBAM powder was used to replace MXene colloidal solution to prepare P(AA‐co‐AM)@PDADMAC hydrogel (referred to as MBAM‐gel) as the control group. After that, the MXene‐gel and Cu wires are encapsulated by polydimethylsiloxane (PDMS) to make a strain sensor.

### DFT Calculation

First‐principles calculations were implemented in Vienna Ab‐Initio Simulation Package (VASP) using density functional theory (DFT) with generalized gradient approximation (GGA) of the form proposed by Perdew‐Burke‐Ernzerhof (PBE). The valence electronic states were expanded on the basis of plane waves. The projector augmented plane wave (PAW) method with a cutoff of 550 eV was used to express the core‐valence interaction. In order to obtain high accuracy through geometry optimization, the Brillouin zone integration was sampled with 4 × 4 × 1 k‐grid mesh. The adsorption energies of different hydrogels for water molecules were calculated by DFT calculations. The adsorption energy (*E_ads_*) was calculated as:

(3) $$E_{ads}=E_{hydrogel-water}\;-E_{hydrogel}-E_{water}$$

Where *E*_hydrogel–water_ is the total energy for the hydrogel with water, *E*_hydrogel_ is the energy for the hydrogel without water, and *E*_water_ is the energy for water.

### Sample Characterization

X‐ray diffraction (XRD) patterns were obtained with a powder diffractometer (DX‐2700B) using Cu Kα radiation of wavelength *k* = 1.54056 Å at 40 kV, 30 mA, and a step scan of 0.03° with 1 s per step. The hydrogels were lyophilised using the freeze dryer (SCIENTZ‐12N). Scanning electron microscopy (SEM) images were obtained using Magellan 400 to obtain high‐magnification images of the treated powders. For the testing of hydrated hydrogels, the water on the surface of the hydrogel needs to be completely removed first, and the entire testing process needs to be completed as quickly as possible. Transmission electron microscopy (TEM) images in this work were obtained using JEOL JSM–2010F. The tensile‐sensing properties were tested using an A11719 Iviumstat electrochemical interface and CHI 760D (CH Instruments Inc., Shanghai, China) electrochemical workstation. At the same time, in order to measure the stability of the strain sensor, cyclic tensile tests were carried out using an electric horizontal test bench (SJX‐500 V, SHANGDU). Tensile and compression tests were performed using a high‐precision micromechanical testing system (Instron5567, America). The water retention of hydrogels was evaluated by exposing them to the environment with the relative humidity (RH) of 40% and the temperature of 25 °C. Fourier transform infrared spectroscopy (FTIR) was performed on a Nicolet IS10 spectrometer to investigate the surface bonding of the composites. The components of the composites were analyzed using X‐ray photoelectron spectroscopy (XPS) (Thermo Escalab 250Xi). Raman spectroscopy (inVia, Renishaw plc, Gloucestershire, United Kingdom) of all MXene materials and composites was performed by using a 532‐nm laser (a spectral resolution ≤2 cm^−1^). Each hydrogel was subjected to low‐field nuclear magnetic resonance (LF‐NMR) analyses (JNM‐ECZ400S/L1, Jeol). The transversal relaxation time (T_2_) were measured at 32 °C with 90° pulse of 12 µs, 180° pulse of 24 µs, scans of 8, echoes of 18000 and echo time of 0.6 ms.

## Conflict of Interest

The authors declare no conflict of interest.

## Supporting information

Supporting Information

## Data Availability

The data that support the findings of this study are available from the corresponding author upon reasonable request.
