# Supplementary material for: MXene‐Induced Flexible, Water‐Retention, Semi‐Interpenetrating Network Hydrogel for Ultra‐Stable Strain Sensors with Real‐Time Gesture Recognition
Source: Adv Sci (Weinh). 2023 Sep 6;10(30):2303922. doi: 10.1002/advs.202303922 (PMC10602575; doi:10.1002/advs.202303922)
Supplement: Supplementary file 1 — Supporting Information [file ADVS-10-2303922-s001.pdf]

ADVANCED SCIENCE
Open Access

Supporting Information

for *Adv. Sci.*, DOI 10.1002/advs.202303922

MXene-Induced Flexible, Water-Retention, Semi-Interpenetrating Network Hydrogel for Ultra-Stable Strain Sensors with Real-Time Gesture Recognition

Lianjia Zhao, Hao Xu, Lingchen Liu, Yiqiang Zheng, Wei Han and Lili Wang**

Supporting Information

MXene-Induced Flexible, Water-Retention, Semi-Interpenetrating Network Hydrogel for Ultra-Stable Strain Sensors with Real-Time Gesture Recognition

Lianjia Zhao†, Hao Xu†, Lingchen Liu, Yiqiang Zheng, Wei Han, and Lili Wang**

Dr. L. J. Zhao, Dr. H. Xu, L.C. Liu, Y. Q. Zheng, Prof. L. L. Wang
State Key Laboratory for Superlattices and Microstructures, Institute of Semiconductors, Chinese Academy of Sciences & Center of Materials Science and Optoelectronic Engineering, University of Chinese Academy of Sciences, Beijing 100083, P.R. China
E-mail: liliwang@semi.ac.cn

Dr. L. J. Zhao, Y. Q. Zheng, Prof. W. Han
College of Physics, State Key Laboratory of Inorganic Synthesis and Preparative Chemistry, International Center of Future Science, Jilin university, Changchun 130012, P.R. China
E-mail: whan@jlu.edu.cn

Keywords: MXene hydrogel, water retention, ultra-stable, strain sensors, machine learning

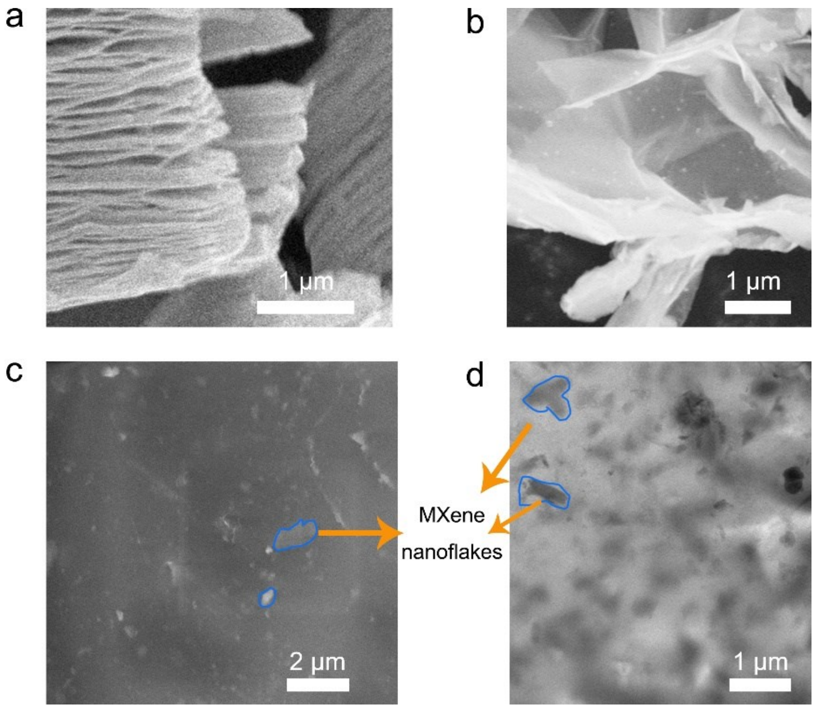

Figure S1. SEM images of (a) multi-layered $Ti_3C_2T_x$ MXene, (b) $Ti_3C_2T_x$ MXene nanoflakes, and (c)hydrated MXene-gel. (d) TEM image of lyophilized MXene-gel.

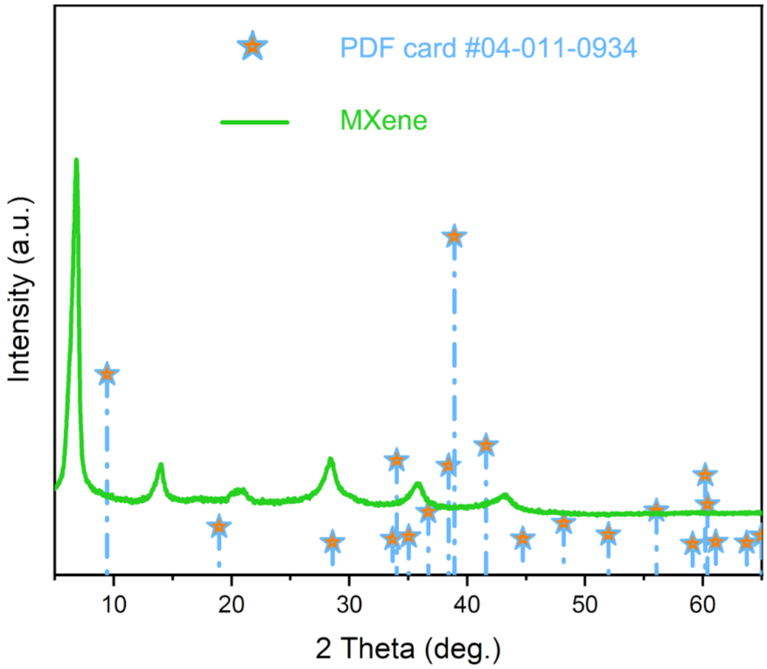

Figure S2. Standard PDF card #04-011-0934 of precursor MAX phase and its etched XRD pattern.

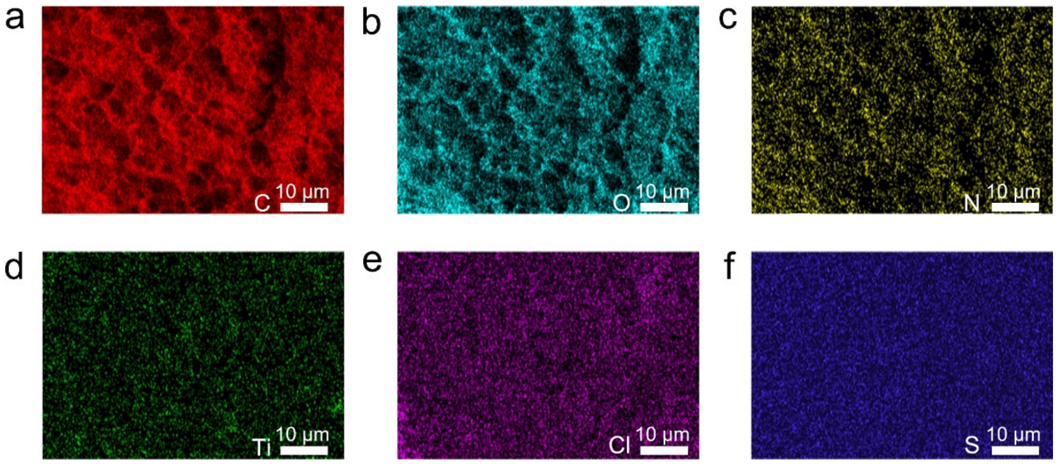

Figure S3. EDS elemental mappings of lyophilized MXene-gel.

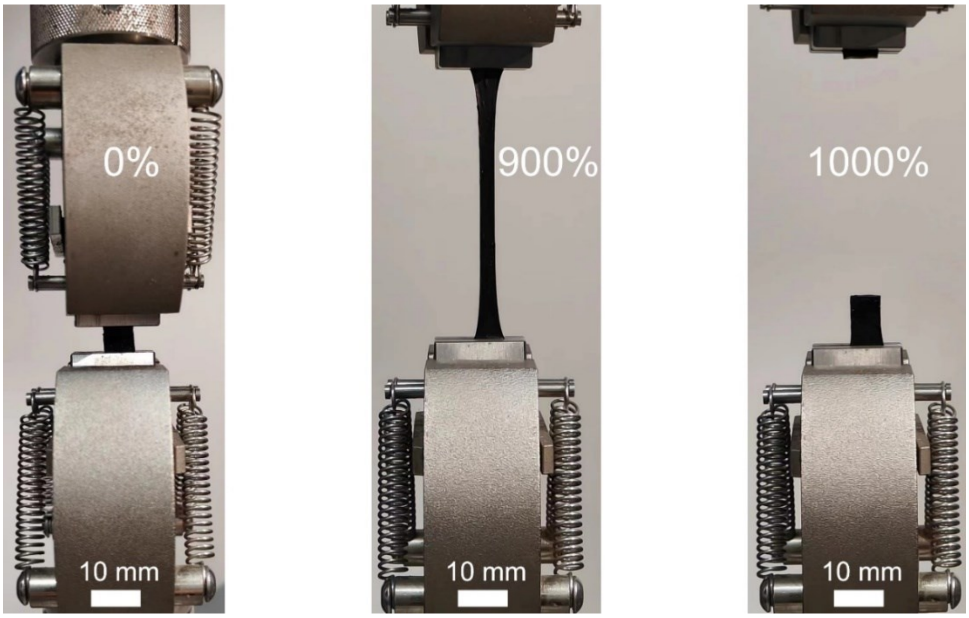

Figure S4. Photos of the machine during testing.

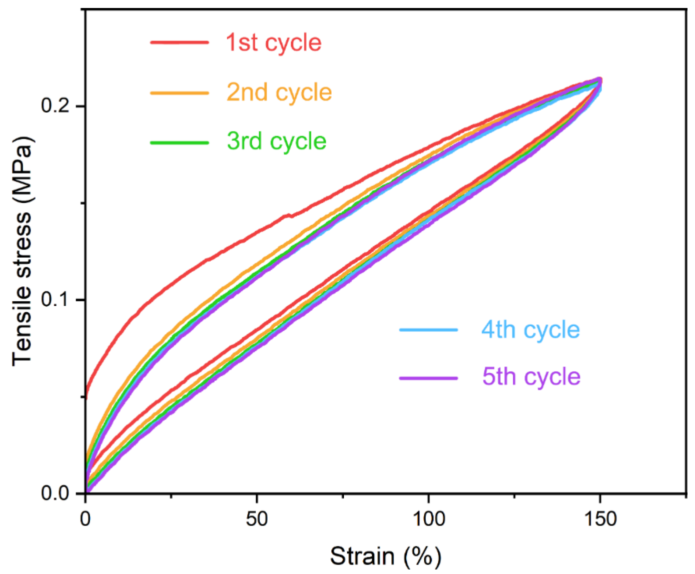

Figure S5. The successive 5 times tensile cycle curves of MBAM-gel.

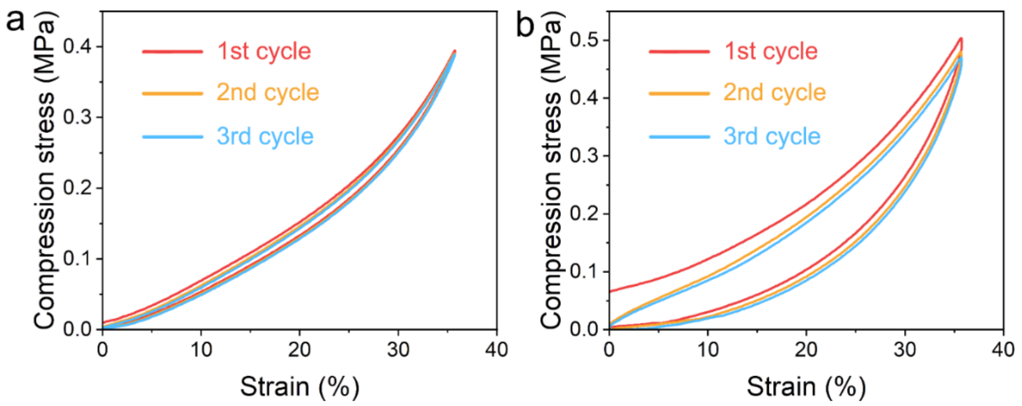

Figure S6. The successive 3 times compression cycle curves of (a) MXene-gel and (b) MBAM-gel.

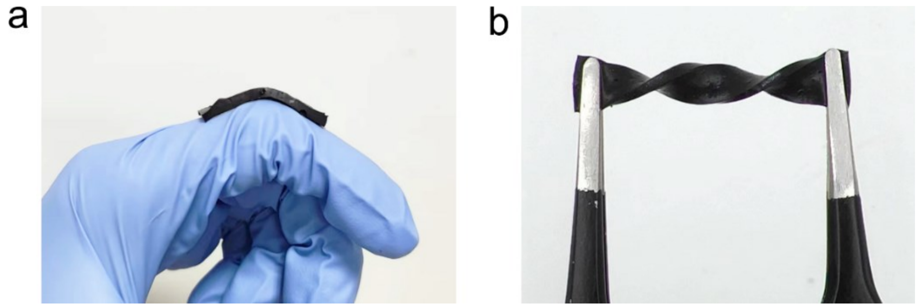

Figure S7. The (a) shape adaptability and (b) twistability of MXene-gel.

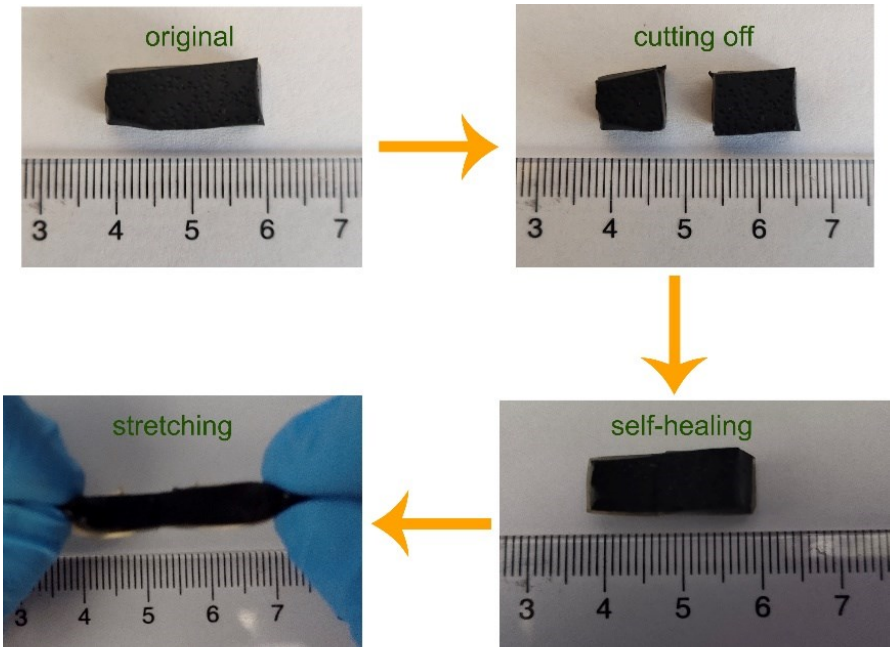

Figure S8. Self-healing properties of MXene-gel.

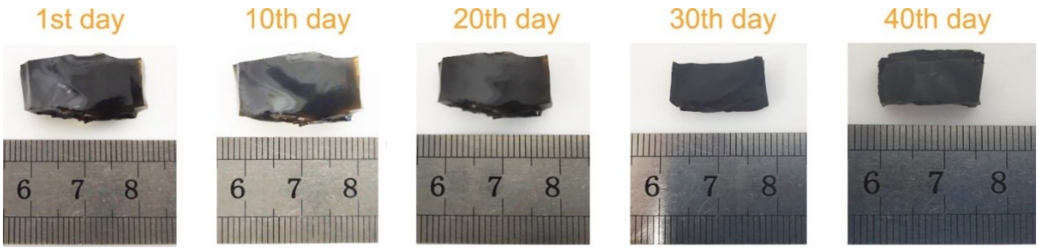

Figure S9. Optical photos of MXene-gel after storage for different days (1-40).

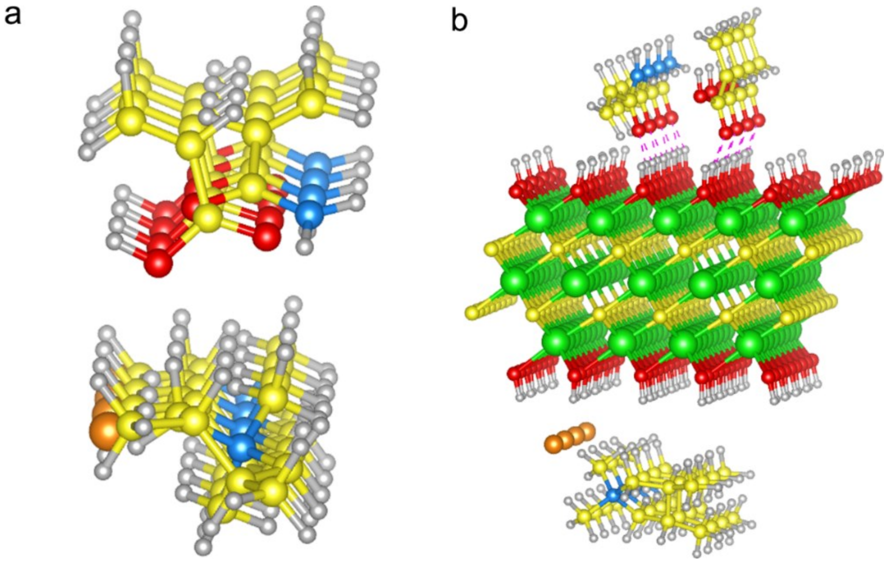

Figure S10. Energy-optimized geometry structure of (a) MBAM-gel and (b) MXene-gel without water based on DFT calculation.

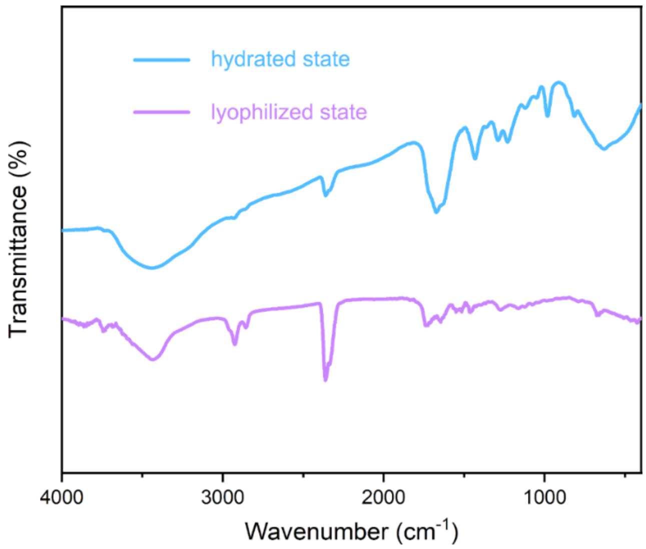

Figure S11. The FTIR spectra of the MXene gel in its original hydrated and lyophilized state.

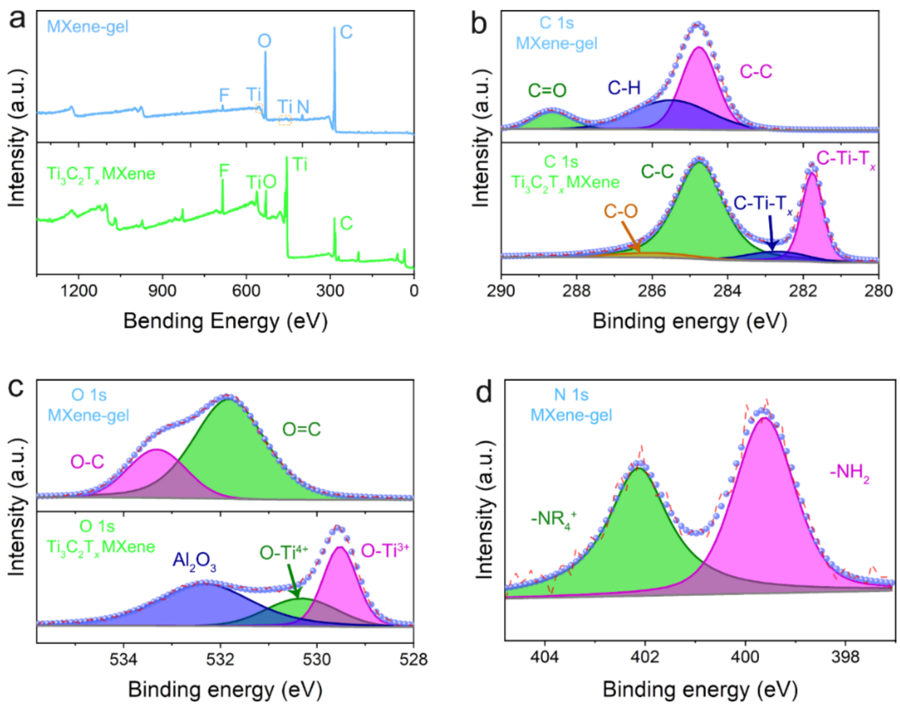

Figure S12. Comparison of the XPS spectra of MXene-gel and MXene. (a) XPS survey spectra of different materials. The XPS spectra analysis of different hydrogels for (b) C 1s, (c) O 1s and (d) N 1s.

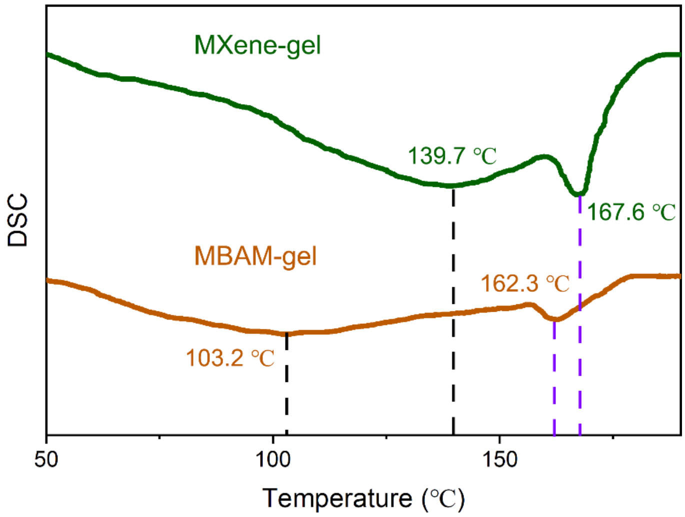

Figure S13. DSC curves of MXene-gel and MBAM-gel.

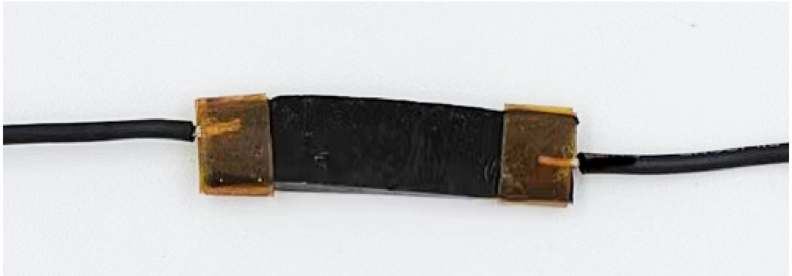

Figure S14. A photograph of the the strain sensor based on MXene-gel.

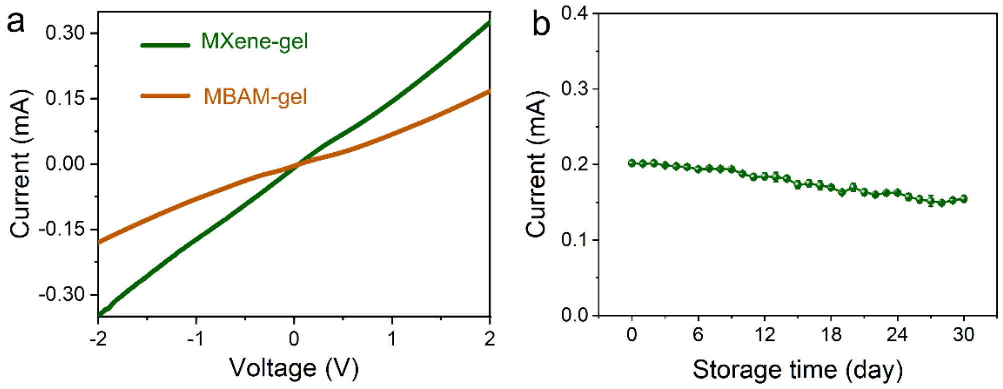

Figure S15. (a) The I−V curves of MXene-gel and MBAM-gel. (b) Current change curve of MXene-gel at different storage days (3 measurements).

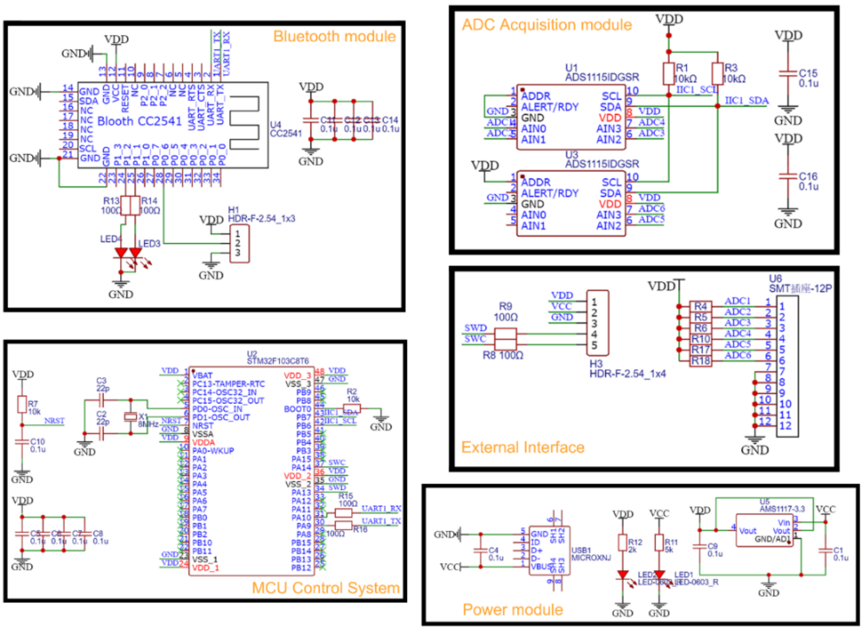

Figure S16. Detailed circuit diagram of each module of the data acquisition system.

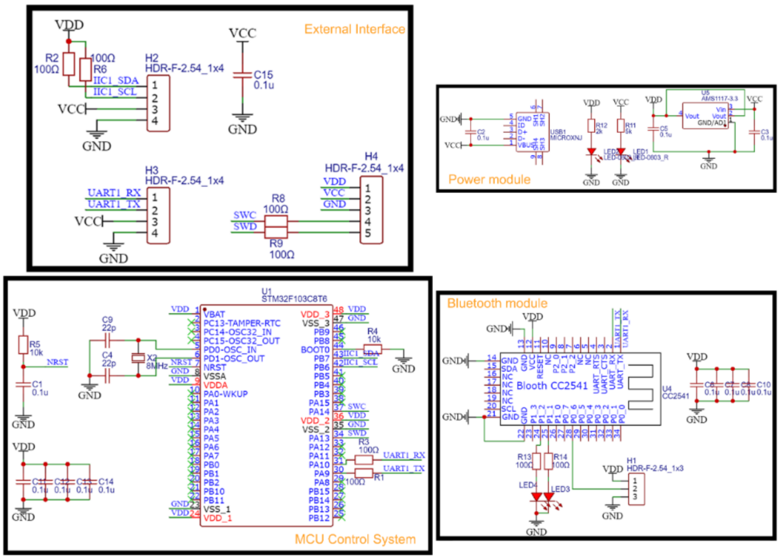

Figure S17. Detailed circuit diagram of each module of the Chinese character display system.

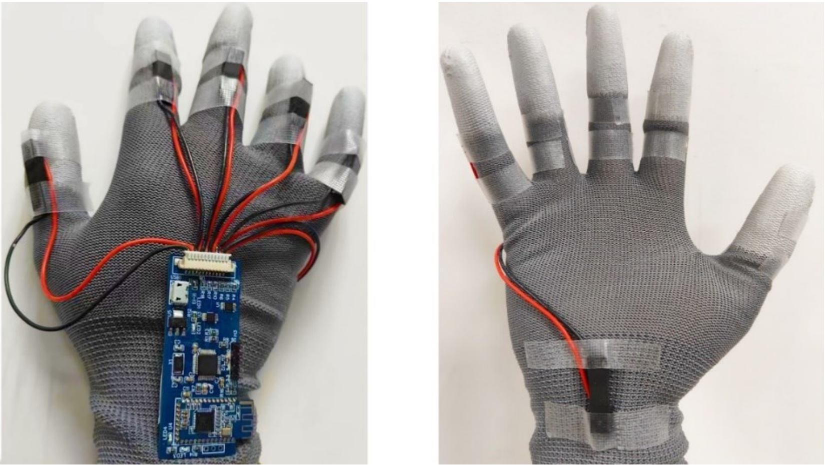

Figure S18. Optical photograph of the portable conversion system.

Table S1. Comparison of mechanical and sensing properties of different hydrogels and strain sensors based on them.

Hydrogel	Tensile strength /MPa	Break strain/ %	Water retention/ day	Sensitivity (%)	T_{res}/s	T_{recov}/s	Reference
P(AA-co-AM)/MXene@ PDADMAC	0.48	1000	40	0.98	0.049	0.066	this work
MXene-PVA/PVP	/	20	1	1.21	/	/	[1]
DPC	1.82	690	/	1.99	260	/	[2]
PVA_{20}-rGO-$B_{1.5}/b_{1.2}$	0.066	367.5	>4	3.79	/	/	[3]
PVA/NaCl	0.7	350	/	2.1	/	/	[4]
PVA/PAM/GA-Zn^{2+}	0.21	914	/	0.209	150	/	[5]
PVA/HPS-PA	9.3	493	/	2.9	220	/	[6]

Abbreviations: PAA, polyacrylic acid; PAM, polyacrylamide; PDADMAC, Poly(diallyldimethylammonium chloride); PVA, polyvinyl alcohol; PVP, polyvinylpyrrolidone; DPC, dual physical cross-linked carboxymethyl cellulose-Fe^{3+}/polyacrylamide; rGO, reduced graphene oxide; GA, gallic acid; HPS, hollow polyaniline spheres; PA, phytic acid; T_{res}, response time; T_{recov}, recovery time.

References

[1] Y. Yi, M. Chiao, K. A. Mahmoud, B. Wang, *Smart Mater. Struct.* **2023**, *32*, 025010.

[2] H. T. Zhang, X. J. Wu, Z. H. Qin, X. Sun, H. Zhang, Q. Y. Yu, M. M. Yao, S. S. He, X. R. Dong, F. L. Yao, J. J. Li, *Cellulose* **2020**, *27*, 9975.

[3] S. P. Dai, X. H. Hu, X. Z. Xu, X. T. Cao, Y. W. Chen, X. S. Zhou, J. N. Ding, N. Y. Yuan, *Synth. Met.* **2019**, *257*, 116177.

[4] Q. Wang, Q. Zhang, G. Y. Wang, Y. R. Wang, X. Y. Ren, G. H. Gao, *ACS Appl. Mater. Interfaces* **2022**, *14*, 1921.

[5] H. Yuan, S. W. Han, S. M. Wang, P. P. Yang, S. W. Li, H.-Y. Mi, C. T. Liu, C. Y. Shen, *Sens. Actuator B-Chem.* **2023**, *379*, 133195.

[6] H. W. Zhou, Z. W. Wang, W. F. Zhao, X. M. Tong, X. L. Jin, X. C. Zhang, Y. Yu, H. B. Liu, Y. C. Ma, S. S. Li, W. X. Chen, *Chem. Eng. J.* **2021**, *403*, 126307.
